# Rehabilitation of Severe Impairment in Motor Function after Stroke: Suggestions for Harnessing the Potentials of Mirror Neurons and the Mentalizing Systems to Stimulate Recovery

**DOI:** 10.3390/brainsci12101311

**Published:** 2022-09-28

**Authors:** Auwal Abdullahi, Thomson W. L. Wong, Shamay S. M. Ng

**Affiliations:** Department of Rehabilitation Sciences, Faculty of Health and Social Sciences, The Hong Kong Polytechnic University, Hong Kong, China

**Keywords:** stroke, mirror neurons, mentalizing system, mental practice, tasks observation, motor function, quality of life

## Abstract

Rehabilitation of severe impairment in motor function following stroke is very challenging. This is because one of the driving forces for recovery of motor function is tasks practice, something this category of patients cannot voluntarily perform. However, it has now been shown that tasks practice can equally be carried out cognitively and through observation of another person’s practice, using techniques known as mental practice and tasks observation, respectively. Mental practice and tasks observation are believed to activate networks of neurons in the brain known as mirror neurons and mentalizing systems to induce recovery. The effectiveness of these techniques has, however, limited evidence at the moment. One possible explanation for this could be the nature of the protocols of these techniques, especially as regards to the intensity of practice. This article proposes ways the potentials of the mirror neurons and mentalizing systems can be harnessed to optimize recovery of severe impairment in motor function using mental practice and tasks observation. The article suggests, among other ways, protocols where tasks observation or mirror therapy are carried out first, and are then followed by mental practice, increasing the number of times the tasks are observed or mentalized, observation of significant others performing the tasks and mental practice of very familiar tasks.

## 1. Introduction

Stroke is a major cause of disability worldwide [1,2]. One of the significant causes of disability following stroke is severe impairment in motor function [3,4]. This is partly because, to date, there seem to be no definite and effective rehabilitation techniques for severe impairment in motor function after stroke. In addition, some of the currently used rehabilitation techniques such as robotic or virtual reality rehabilitation can be very costly; hence limiting their utilization even in the technologically advanced countries of the world. However, according to the Alma-Ata declaration, provision of healthcare is a basic human right irrespective of one’s position in the society or socioeconomic status [5]. Thus, finding cost-effective rehabilitation techniques for severe impairment in motor function is warranted.

Accordingly, two popular rehabilitation techniques that seem to offer some hope for patients with severe impairment in motor function are tasks observation and mental practice, sometimes known as motor imagery [6,7]. Tasks observation is a rehabilitation technique whereby a patient observes physical performance of tasks by a second person with the goal of having the cortical activation that occurred in the brain of the second person during the tasks performance mirrored in similar brain areas of the patient [8,9]. On the other hand, mental practice is defined as the cognitive rehearsal of functional movements in the absence of actual physical performance [10,11]. This similarly helps to engage and stimulate areas of the patient’s brain responsible for the control of movement as in during physical performance [12,13]. Activation of brain areas responsible for control of movement during tasks observation and mental practice is said to be possible due to the presence of specialized networks of neurons known as the mirror neurons and the mentalizing systems in the human brain [14]. The mirror neurons and the mentalizing systems are activated through actions performed physically, mentally or emotionally [6,10,15]. In fact, the primary motor (M1) is involved in the cognitive process of movement execution, in addition to generating the impulses required for execution of movement [16,17]. Thus, mental practice or motor imagery can stimulate or activate it.

However, available evidence still suggests that these techniques seem not to be significantly superior to traditional or conventional therapy [18]. This could be due to inadequate intensity of the tasks performed during the interventions. For example, during mental practice, the amount or intensity of practice is not clear, except for mention of the number of minutes the patients spent carrying out the tasks [19]. The aim of this paper is to therefore discuss the mirror neurons and mentalizing systems and how we can harness their potentials by optimizing delivery of interventions such as the tasks observation, mental practice and mirror therapy to optimize recovery of severe impairment in motor function following stroke. This will be done through provision of suggestions on how tasks observation and mirror therapy are first carried out, and then followed by mental practice, increasing the number of repetitions of the tasks observation and mental practice, observing loved ones or significant others performing the tasks and practicing tasks that the patient is used to during mental practice and tasks observation.

## 2. The Mirror Neurons and the Mentalizing Systems

Mirror neurons and mentalizing systems have distinct functional roles [20]. The mirror neuron system is formed by several areas of the brain, which include the posterior inferior frontal gyrus, the rostral part of the inferior parietal cortex, the dorsal premotor cortex and the primary motor cortex [15,21,22]. The main role of these brain areas are coding for action execution and perception, with the areas in the frontal gyrus coding for goals of the action and the areas in the parietal cortex coding for the means of the action [23,24,25].

**Figure 1:**
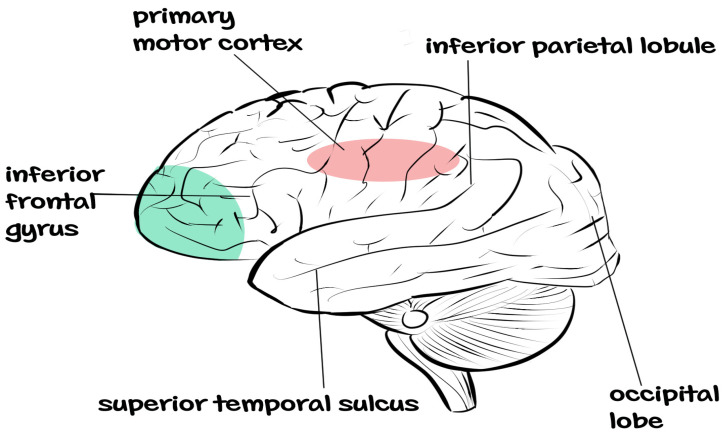
Anatomy of the mirror neuron system (Adapted from Rajmohan and Mohandas [30]).

The above-mentioned specializations in the roles of the different brain areas have been argued to be what is responsible for the human ability to imitate the actions of others by matching both the means of action coded by the parietal cortex and the goals of action coded by the brain areas in the frontal gyrus [26,27,28]. Consequently, observing a second person performing tasks or observing one’s actions will result in the activation of the corresponding areas in the patient’s brain that were activated in the second person’s brain during the tasks performance [23,29]. This provides a hopeful leverage to set the brain on the road to recovery in severe cases of impairment in motor function following stroke. See Figure 1 for the anatomy of the mirror neuron system.

On the other hand, the areas of the brain that form the mentalizing system are the posterior superior temporal sulcus, extending into the temporo-parietal junction, posterior cingulate cortex, the precuneus and the dorsomedial prefrontal cortex [30,31,32,33,34]. These different areas that form the mentalizing system are activated during different situations. For instance, during theory of mind tasks and observation of social interactions, the areas that are usually activated are the posterior cingulate cortex and the precuneus [32,33,34].

**Figure 2:**
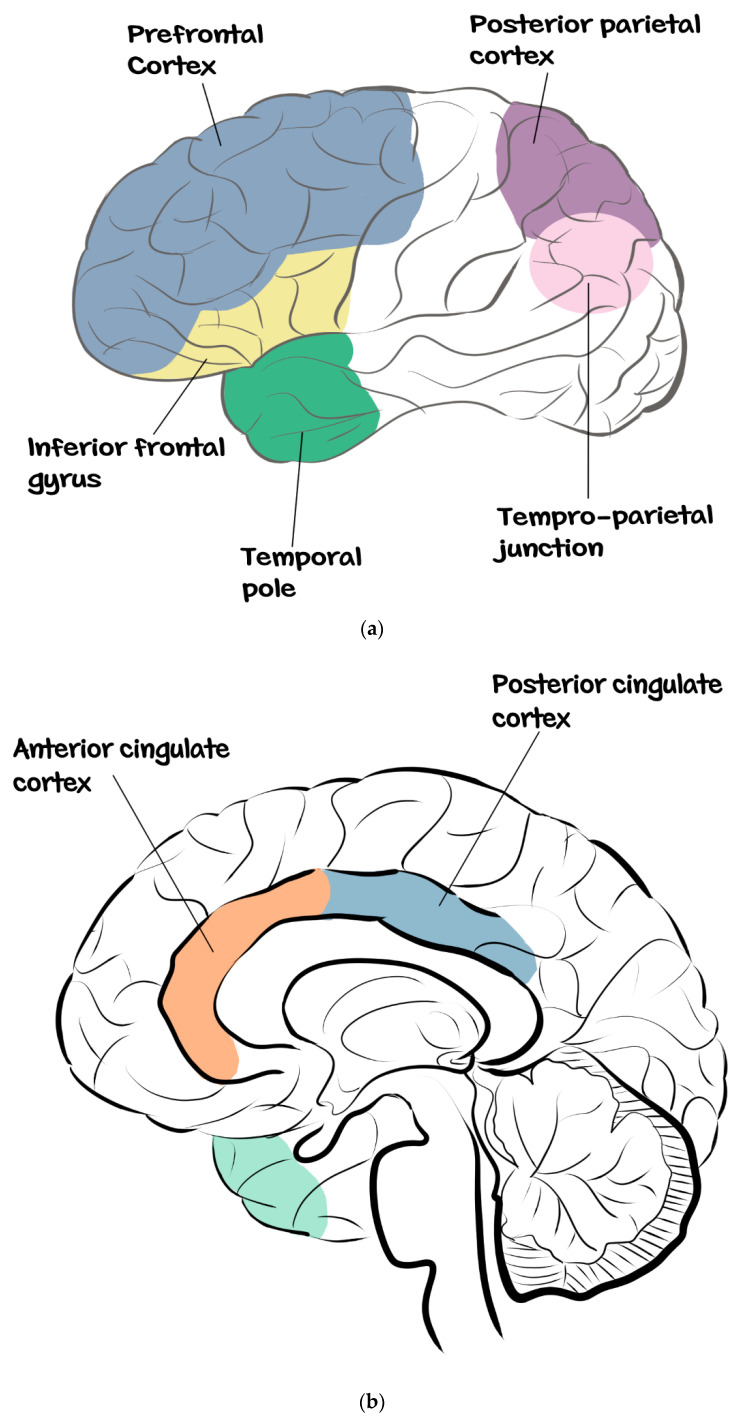
(**a**) Dorsal view of the anatomy of the mentalizing system. (**b**) Ventral view of the anatomy of the mentalizing system (Adapted from Monticelli et al. [40]).

Similarly, the temporo-parietal junction is involved in mind reading and plays an important role in the attribution of external agency [35,36,37]. Interestingly, this set of regions corresponds to the ‘‘default mode network’’ that shows a sustained activity during self-referential processing [38]. Thus, the mentalizing system appears to be involved in both representation of others’ mental states and in self-referential processes [39]. Consequently, imagining oneself performing tasks will result in the activation of this system of neurons known as the mentalizing system in the patient’s brain. See Figure 2a,b for the dorsal and ventral views of the anatomy of the mentalizing system.

## 3. Suggestions for Harnessing the Potentials of Mirror Neurons and the Mentalizing Systems to Stimulate Recovery

### 3.1. Tasks Observation, Followed by Mental Practice

Tasks observation is a multisensory approach encompassing motor somatosensory and cognitive rehabilitation, whereby patients are made to observe performance of tasks practice by a second person either in the real world or in a video [9,40,41,42]. It has been reported to improve motor function, activities of daily living and cortical activation in patients with stroke [9,43]. On the other hand, mental practice is an intervention that encompasses cognitive rehearsal of functional tasks and activities people carry out in their daily lives [9,10,14,44]. It is effective at improving activity limitation following stroke [19].

Both tasks observation and mental practice are believed to have a common neuroplastic pathway, the mirror neurons and the mentalizing systems [10,14,32,45]. However, it is often thought that it is difficult to ascertain whether or not patients actually mentalize the tasks during mental practice. Consequently, since tasks observation and mental practice have a common neuroplastic pathway, the mirror neuron and the mentalizing systems, it makes clinical sense to combine these interventions as one in a single protocol. Thus, when patients observe a task performance, they should immediately be made to mentalize it. That way, the uncertainty around whether patients actually perform mental practice or not could be overcome.

In addition, sometimes the mirror neurons and the mentalizing systems can be engaged simultaneously [46]. Thus, patients should be made to perform mental practice immediately after they observe a second person or themselves perform the tasks they are required to perform with the affected limb. The combination of tasks observation and mental practice has been shown to improve motor outcomes after stroke [47,48]. This seems to suggest the potential positive effect of combining the two interventions, especially when the task observed is immediately followed by mental practice of the same task.

### 3.2. Observing the Task Performance of Familiar Faces and Mentalizing Very Familiar or Everyday Tasks

Familiarity tends to modulate both the mirror neurons and the mentalizing systems [49]. Consequently, use of familiar faces during tasks observation and mentalizing familiar or everyday tasks may provide an additional advantage. This is because the mentalizing process can be triggered by many cues; however, conspecific cues are usually the most important triggers [50]. Thus, using a significant other such as a spouse or the beloved children or grandchildren as the subjects the patient will observe performing tasks, may help activate the mirror neurons system more efficiently. Similarly, the mental practice of familiar or everyday tasks may help to ease the process of activating the mentalizing system. This is because mental practice involves cognitive or mental representation of tasks [51]. Memories for such tasks are already stored in the brain, which makes them easier to be retrieved when needed.

### 3.3. Mirror Therapy, Followed by Mental Practice

Mirror therapy is a rehabilitation technique that involves the use of visual stimulation to create the illusion of movement of the affected limb [52,53]. The intervention is carried out by placing a mirror in the mid-sagittal plane of the patient. Therefore, as the unaffected limb is moved, it is reflected in the mirror as if it were the affected limb that moved [54]. This will in turn activate the mirror neuron system in the patient. In addition, it is reported to improve the use of the limb in functional activities required for daily living [55].

From the foregoing, mirror therapy seems to be similar to tasks observation in a sense. Thus, it is not surprising that mirror therapy seems to share the same neurological pathway with tasks observation, in that it may also activate the mirror neuron system [56]. Therefore, to help fortify the potential effects of the mirror therapy on mirror neurons, it is followed immediately by mental practice.

### 3.4. The Role of Music Therapy

Adding music therapy or sound during tasks observation before mental practice could provide additional benefit. This is because there is a group of mirror neurons known as the audio-visual neurons that respond to sound [57,58,59]. In addition, music has a distinct ability to evoke memory [60]. Consequently, it may evoke the patient’s memory of how the affected limb was used previously for activities of daily living, thus mimicking mental practice in a way. When music therapy is therefore followed by mental practice, activation of the mirror neuron and mentalizing systems could be further enhanced.

### 3.5. High Repetition of Tasks Observation and Mental Practice

The current protocols for tasks observation and mental practice seem to be not very clear in terms of intensity or dose of tasks practice. In fact, the intensity seems to be inadequate [19]. However, even with the active physical performance of tasks practice, high repetition of tasks practice is required for motor recovery [61,62]. Similarly, for tasks observation and mental practice, techniques that are considered passive forms of active repetitive tasks practice, the higher repetition principle should apply even more.

### 3.6. Combining Tasks Observation and Mental Practice with other Interventions

Hybrid therapy is gaining more ground in neurorehabilitation. It means combination of more than one rehabilitation technique to help optimize recovery [63]. An example of hybrid therapy can be combining neurodevelopmental technique (NDT) with brain stimulation such as transcranial direct current stimulation. In a previous study, a combination of robotic-assisted therapy with constraint-induced movement therapy or bilateral arm training improves motor function and functional goals [63]. Thus, for the rehabilitation of severe impairment in motor function, tasks observation and mental practice can be combined with techniques such as peripheral neuromuscular stimulation, brain stimulation or robotic rehabilitation.

### 3.7. Environment for Rehabilitation Should Represent Real World Situation or Environment for the Patient

When designing rehabilitation that is aimed at utilizing the mirror neurons and the mentalizing system, the environment, for example, the laboratory or clinic where the intervention will be carried out, should represent a real world situation or environment. For instance, the laboratory should simulate the sitting room or balcony of the patient’s residence. This is because carrying out tasks in the real world is a significant predictor of the patient’s ability to carry out a high-dose of massed practice [64]. High intensity of practice is a precursor to recovery of motor function [62].

## 4. Conclusions

Mirror neurons and mentalizing systems offer an excellent opportunity for the stimulation of recovery of severe impairment in motor function following stroke. In particular, their potentials could be optimized when tasks observation or mirror therapy is followed immediately by mental practice of observed tasks, and when music therapy is employed during tasks observation. Similarly, use of high-dose of repetitions of tasks during mental practice, use of familiar faces during tasks observation and using a real-life environment or situation during tasks observation and mental practice may equally help to optimize recovery. However, prospective clinical studies to test our propositions are warranted.

## Data Availability

Not applicable.
